# The Niagara Medical College

**Published:** 1884-09

**Authors:** 


### The Niagara Medical College.

The second annual session of this institution opens October 1st under most flattering auspices. The opposition, received from numerous enemies connected with an institution with which the promoters of the new school have never designed to enter into competition, have so widely advertised the college among the profession that whatever anticipations of success may have been held are sure to be more than realized. This is especially encouraging, and demonstrates that the current sentiment of the profession throughout the country is in accord with the higher standard, to which the college is fully committed, both by its charter and by the oft-repeated declarations of its trustees and faculty. Thorough didactic and clinical instruction will be given, and students, arranged in classes, according to their proficiency and the period of their pupilage, will receive the most complete drilling which it is possible for its corps of instructors to give. The success of this course cannot be doubted, and the results will be felt both here and elsewhere. As to the facilities offered to students, no institution outside of the great medical centres will be able to surpass those provided by the Niagara school; and whatever diplomas may be granted will not be the exponent of so much time ostensibly devoted to study by the recipient, but, rather, the certificate for qualifications, upon which an able independent body of examiners have sat in judgment.

We ask the profession to examine the methods and principles upon which this institution is based. We anticipate a warm endorsement, and we are able to give assurance that the college will warrant the fullest confidence that can be bestowed.

